# Incidence, mortality, and lethality of hospitalizations for community-acquired pneumonia with comorbid cardiovascular disease in Spain (1997–2015)

**DOI:** 10.1186/s12879-020-05208-y

**Published:** 2020-07-06

**Authors:** Loreto Arias-Fernández, Ruth Gil-Prieto, Ángel Gil-de-Miguel

**Affiliations:** 1 Rey Juan Carlos University, Madrid, Spain; 2 Area of Preventive Medicine & Public Health, Rey Juan Carlos University, Avda. Atenas s s/n, 28922 Alcorcón (Madrid), Spain

**Keywords:** Pneumonia, Hospitalization, Cardiovascular disease

## Abstract

**Background:**

The probability of hospitalization in patients suffering from community-acquired pneumonia (CAP) with an underlying comorbidity, such as a cardiac pathology, is 73-fold higher than that in CAP patients without a comorbidity. Although previous studies have investigated patients with cardiac events and pneumonia, they have not studied the burden of disease in depth at the population level. The objective of this study is to provide population-level data on patients ≥60 years old who were hospitalized with pneumonia with comorbid cardiovascular disease (CVD) in Spain over a period of 19 years (1997–2015).

**Methods:**

This is a retrospective study based on a minimum basic data set (MBDS). The following variables were collected: age, sex, re-admission (yes/no), hospital stay (days), and other diagnoses. Hospitalization rate (per 100,000 inhabitants), mortality rate (per 100,000 inhabitants), and lethality rate (%) were obtained, and the 95% confidence interval of each rate was calculated. Analyses were stratified by age (categorized into 4-year intervals), sex, and year of admission. Differences were assessed for significance with the chi-squared test for proportions and the Poisson model for rates. Logistic regression was run with in-hospital survival as the dependent variable and sex, age, year of admission, and re-admission (yes/no) as the independent variables. The level of significance was *p* < 0.005.

**Results:**

The total number of patients ≥60 years old hospitalized for pneumonia with comorbid CVD was 99,346. The rates of hospitalization, mortality, and lethality increased significantly with age over the 19 years. Men had higher rates of hospitalization and mortality. The probability of a patient with CAP and CVD dying was correlated with male sex, older age, hospital re-admission, and having been hospitalized earlier in the study period.

**Conclusions:**

Community-acquired pneumonia with comorbid cardiovascular disease continues to be a major cause of hospitalization in Spain, especially in the elderly population, making it necessary to develop more preventive strategies for this group of patients.

## Background

Lower respiratory tract diseases are the fourth leading cause of death in the world [1]. Pneumococcal pneumonias accounted for more than 20% of lower respiratory-related diseases in 2013 [2] and represented the third-leading cause of years of life lost adjusted for disability [3]. This problem is particularly pertinent in people ≥65 years of age [4], for whom there is also a higher health expenditure when treating community-acquired pneumonia (CAP) [5]. The probability of hospitalization in patients suffering from CAP with an underlying comorbidity in the form of a cardiac, respiratory, or metabolic pathology is 73-fold higher than that in CAP patients without a comorbidity [6].

The main causes of mortality worldwide are ischaemic cardiomyopathy and stroke [7]. In Spain, diseases of the circulatory system were the main cause of death in 2016 [8]. Cardiac events in patients with CAP are significantly associated with in-hospital mortality [9], a longer hospital stay, and more severe CAP [10]. Furthermore, CAP can exacerbate or cause cardiovascular complications [11], and cardiac markers are predictors of mortality in patients hospitalized for CAP [12]. In addition to CAP in general, pneumococcal pneumonia confers an increased risk of having a cardiac event [13]. Furthermore, cardiovascular disease (CVD) is a risk factor for CAP [14, 15].

Population databases are useful for establishing disease burden because they have a complete and standardized collection of hospitalization records. The Spanish National Discharge Database has proven to be a reliable tool for studying the hospitalization load for CAP [16, 17].

Previous studies have investigated patients with cardiac events and pneumonia, but the burden of disease at the population level has not been studied in depth [18]. The objective of this study is to provide population data on the hospitalization burden of CAP with comorbid CVD in Spain over a period of 19 years (1997–2015).

## Methods

This is a retrospective study based on a minimum basic data set (MBDS) and supported by the Ministry of Health of Spain. This system used the International Classification of Diseases (ICD-9-CM) and covered approximately 98% of public hospitals and some private hospitals. The public health care system covers 99.5% of the Spanish population, making it a representation of people hospitalized at the state level. The MBDS has been validated with regard to data quality and overall methodology by the Ministry of Health of Spain [19, 20].

All hospital admissions of patients aged 60 years and older for all causes, with pneumonia (ICD-9-CM 480–486) in the principal diagnostics position at admission and at least one diagnosis of cardiovascular disease, occurring between 1st January 1997 and 31st December 2015 in Spain were obtained from the MBDS. The eligible CVDs are listed in Table 1 and were based on their use by other authors [21].

**Table 1:** Definition of cardiovascular diseases used according to the ICD 9 CM

Acute rheumatic fever (390–392)	
390:	Rheumatic fever without involvement of the heart
391:	Rheumatic fever with involvement of the heart (391.0–391.9)
392:	Rheumatic chorea (392.0–392.9)
Chronic rheumatic heart disease (393–398)	
393:	Chronic rheumatic pericarditis
394:	Mitral valve diseases (394.0–394.9)
395:	Aortic valve diseases (395.0–395.9)
396:	Diseases of the mitral and aortic valves (396.0–396.9)
397:	Diseases of other structures of the endocardium (397.0–397.9)
398:	Other rheumatic heart disease (398.0–398.99)
Hypertensive disease	
402:	Hypertensive heart disease (402.0–402.91)
403:	Hypertensive kidney disease (403.0–403.9)
404:	Hypertensive kidney and heart disease (404.0–404.9)
Ischaemic heart disease (410–414)	
410:	Acute myocardial infarction (410.0–410.9)
411:	Another form of acute and subacute ischaemic heart disease (411.0–411.89)
412:	Old myocardial infarction
413:	Angina pectoris (413.0–413.9)
414:	Other forms of chronic cardiac ischaemic disease (414.0–414.19)
Pulmonary circulation disease (415–417)	
415:	Acute pulmonary heart disease (415.0–415.19)
416:	Chronic pulmonary heart disease (416.0–416.9)
417:	Other pulmonary circulation diseases (417.0–417.9)
Other forms of heart disease	
420:	Acute pericarditis (4200–420.99)
421:	Acute and subacute endocarditis (421.0–421.9)
422:	Acute myocarditis (422.0–422.99)
423:	Other pericardial diseases (423.0–423.9)
424:	Other endocardial diseases (424.0–424.99)
425:	Cardiomyopathy (425.0–425.9)
428:	Heart failure (428.0–428.9)
429:	Complications and poorly defined descriptions of heart disease (429.0–429.9)
Cerebrovascular disease	
430:	Subarachnoid Haemorrhage
431:	Intracerebral haemorrhage
432:	Other nonspecific intracranial haemorrhages (432.0–432.9)
433:	Stenosis and occlusion of the pre-cerebral arteries (433.0–433.9)
434:	Occlusion of the cerebral arteries (434.0–434.9)
436:	Acute but poorly defined cerebrovascular disease
437:	Other and poorly defined cerebrovascular diseases (437.0–437.9)
438:	Late effects of cerebrovascular disease (438.0–438.9)
Disease of the arteries, arterioles and capillaries	
440:	Atherosclerosis (440.0–440.9)
441:	Aneurysm and aortic dissection (441.0–441.9)
444:	Arterial embolism and thrombosis (444.0–444.9)
446:	Polyarteritis nodosa and related conditions (446.0–446.7)
447:	Other alterations of the arteries and arterioles (447.0–447.9)

The following variables were collected: age, sex, re-admission (yes/no), hospital stay (days), other diagnoses (up to 13 additional diagnostic positions) and outcome (discharge/death). Re-admission was defined as “being re-admitted to the hospital with the same principal diagnostic code at admission within 30 days of being discharged.” Absolute (n) and relative (%) frequencies were calculated for the number of hospitalizations per year, sex, re-admission, and death. The mean and standard deviation (SD) of age, hospital stay, and number of diagnoses were calculated. The rate of hospitalization (per 100,000 inhabitants), in-hospital mortality rate (per 100,000 inhabitants), and in-hospital lethality rate (% of hospitalizations) were calculated, and corrected population data from the municipal records, extracted from the National Institute of Statistics (https://ine.es/dyngs/INEbase/en/categoria.htm?c=Estadistica_P&cid=1254734710984, accessed on 28 April 2020), were used as the denominator for the hospitalization and mortality rates. The 95% CI of each rate was calculated. The results were stratified by sex, age (categorized into 4-year intervals), and year of admission. Significant differences were analysed with the chi-squared test for proportions and the Poisson model for rates during the period studied. Logistic regression was performed with in-hospital survival as the dependent variable and sex, age, year, and re-admission as independent variables. The level of significance was *p* < 0.05.

For the statistical analysis, we used SPSS v.22 software (IBM Corp., New York, USA).

The personal information of each subject was delivered to the researchers anonymously, impeding the traceability of the subject, in strict adherence to Spanish and European legislation. The present project received a waiver from the local ethics committee, Comité de Ética de la Investigación de la Universidad Rey Juan Carlos, which ruled that no formal ethics approval was required.

## Results

The total number of patients ≥60 years old with CVD who were hospitalized for pneumonia was 99,346 in the study period. A total of 60.7% were male, and the average age was 79.80 years (SD: 8.26), which was distributed as follows: 60–64 years, 4.5%; 65–69 years, 7.9%; 70–74 years, 13.7%; 75–79 years, 20%; 80–84 years, 22.9%; and ≥ 85 years, 30.9%. The average length of hospital stay was 11.23 days (SD: 10.5), and 12.6% were re-admitted 30 days after discharge. Overall, 13.8% of patients died, reaching 17.7% of patients when counting re-admissions. A total of 75.7% of patients had pneumococcal pneumonia (ICD-9 CM: code 481) in the principal diagnostic position. The mean number of diagnoses present was 8.47 (3.03) per patient.

The annual global hospitalization rate due to CAP with comorbid CVD was 55.27 (95% CI: 54.92–55.61) hospitalizations per 100,000 inhabitants, reaching 30,739 hospitalizations in the age group of ≥85 years. The annual global in-hospital mortality rate was 32.71 (95% CI: 32.16–33.26) deaths per 100,000 inhabitants, reaching 656.50 deaths per 100,000 inhabitants in those over 85 years. The global annual in-hospital lethality rate was 13.81% (95% CI: 13.60–14.02), with the highest rate at 19.53% in the age group older than 85 years.

Table 2 shows the rates of hospitalization and mortality per 100,000 inhabitants and the lethality rate (%) by age and sex. Rates increase significantly with age. The mortality and hospitalization rates were significantly higher in men in all age groups (*p* < 0.001), although the lethality rate showed no sex differences.

**Table 2:** HR, MR, and LR distribution by sex and age group

	Hospitalization rate/100,000 inhabitants (95% CI)	Mortality rate/100,000 inhabitants (95% CI)	Lethality rate/% (95% CI)
Men			
*60–64 years*	16.5 (15.94–17.07)	1.24 (1.08–1.39)	7.49 (6.59–8.39)
*65–69 years*	32.07 (31.24–32.89)	2.71 (2.47–2.95)	8.44 (7.72–9.16)
*70–74 years*	62.52 (61.27–63.77)	6 (5.61–6.39)	9.60 (9.01–10.18)
*75–79 years*	108.72 (106.87–110.57)	12.81 (12.18–13.45)	11.78 (11.24–12.33)
*80–84 years*	173.25 (170.33–176.16)	24.55 (23.46–25.65)	14.17 (13.59–14.76)
*≥ 85 years*	283.23 (278.66–287.8)	56.24 (54.2–58.27)	19.85 (19.21–20.50)
*Total*	76.64 (76.03–77.25)	10.26 (10.04–10.48)	13.39 (13.11–13.66)
Women			
*60–64 years*	5.46 (5.15–5.77)	0.33 (0.26–0.41)	6.09 (4.72–7.46)
*65–69 years*	10.16 (9.72–10.6)	0.79 (0.67–0.91)	7.75 (6.6–8.9)
*70–74 years*	21.31 (20.65–21.97)	1.9 (1.7–2.1)	8.92 (8.03–9.8)
*75–79 years*	39.84 (38.88–40.8)	4.28 (3.97–4.6)	10.75 (10.01–11.5)
*80–84 years*	72.91 (71.42–74.4)	10.07 (9.51–10.62)	13.81 (13.1–14.51)
*≥ 85 years*	140.15 (137.98–142.32)	26.96 (26.01–27.91)	19.24 (18.63–19.85)
*Total*	38.65 (38.26–39.03)	5.59 (5.44–5.74)	14.46 (14.12–14.81)
Age groups			
*60–64 years*	10.78 (10.47–11.1)	0.77 (0.68–0.85)	7.13 (6.37–7.88)
*65–69 years*	20.47 (20.01–20.92)	1.69 (1.56–1.82)	8.26 (7.65–8.87)
*70–74 years*	39.93 (39.27–40.6)	3.75 (3.55–3.96)	9.4 (8.91–9.89)
*75–79 years*	68.98 (68.03–69.94)	7.89 (7.57–8.22)	11.44 (11–11.88)
*80–84 years*	111.25 (109.8–112.69)	15.6 (15.06–16.14)	14.03 (13.57–14.48)
*≥ 85 years*	184.88 (182.82–186.95)	36.11 (35.2–37.02)	19.53 (19.09–19.98)
*Total*	55.27 (54.92–55.61)	7.63 (7.5–7.76)	13.81 (13.6–14.02)

**Fig. 1:**
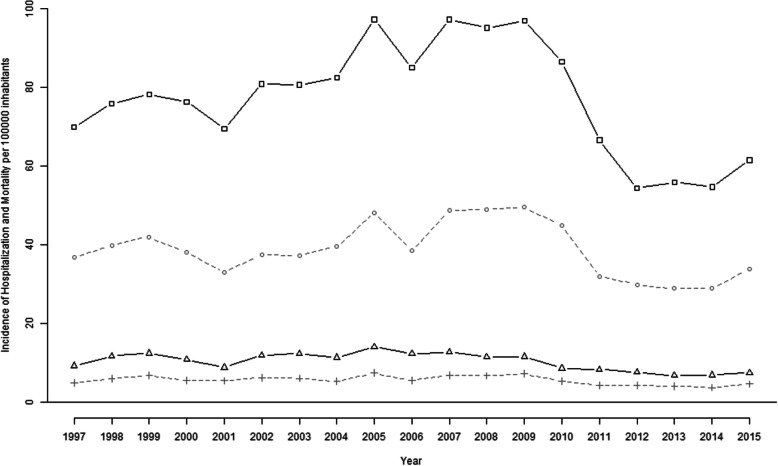
Hospitalization Rate and Mortality Rate by Sex and Year

Figure 1 shows the time trend of incident hospitalizations due to CAP and its associated mortality rates stratified by sex. Using a linear approximation in Poisson rate regression, the declining trend in hospitalizations was similar for men and women (men: -2.3% (RR 0.98, 0.975–0.978) and women − 2.2% (RR = 0.98, 0.976–0.980) per year). A similar trend was observed for the mortality rate. Using a linear approximation in Poisson rate regression, the declining trend in mortality was 4% for men (RR 0.96, 0.956–0.964) and 3.4% for women (RR = 0.97, 0.961–0.970) per year.

**Fig. 2:**
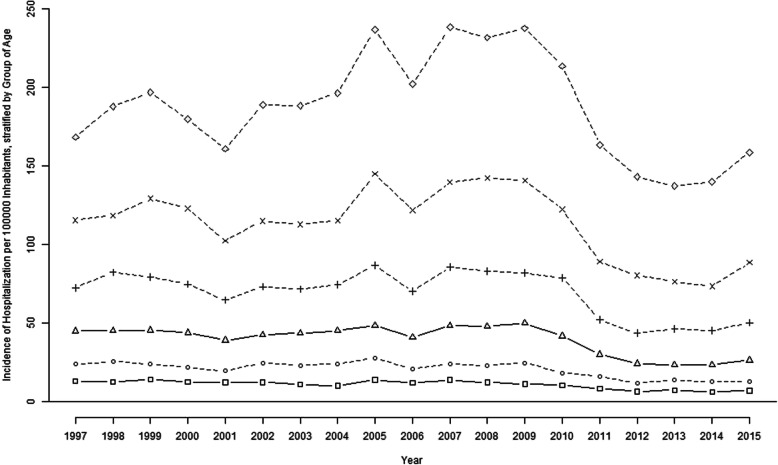
Hospitalization Rate by Age Group and Year

Figure 2 shows the hospitalization rate according to age group over the 19-year study period. An increase was seen in all age groups between 2007 and 2010, then a decrease until 2014, followed by an increase again in 2015. Using a linear approximation in Poisson rate regression, the declining trend in hospitalizations was significantly modified by age group (p_INT_ < 0.001), with lower reductions in hospitalizations by year as age increased, ranging from a − 3.5% reduction in the youngest age group [60,65) to a − 1.3% reduction in the oldest age group [85+).

Death during hospitalization was significantly associated with age in years (OR = 1.05, 95% CI: 1.41–2.19) and readmission to the hospital within 30 days (OR = 1.70, 95% CI: 1.62–1.79); moreover, it was inversely associated with being female (OR = 0.94, 95% CI: 0.90–0.98) and the year of hospitalization (OR = 0.97, 95% CI: 0.97–0.98).

## Discussion

This study evaluated the burden of hospitalizations for pneumonia with CVD at the population level in patients older than 60 years in Spain and confirmed the high burden of the disease in this risk group. Each year, on average, 55 out of 100,000 inhabitants older than 60 years of age were hospitalized for CAP with a comorbid CVD diagnosis in Spain. Using a linear time trend, a 2% reduction in hospitalization rates could be observed, a tendency more pronounced in the younger age group of 60–65-year-olds.

In the period described, there were many hospitalizations for CAP with comorbid CVD in people over 60 years of age, and the group older than 85 made up almost a third of them. This is important because the population of Spain is ageing overall, and its life expectancy is increasing. Hospitalized older people also present with complications that negatively affect their health and later recovery [20], especially patients with risk factors such as CVD. The average hospital stay was 11 days, and the average length of stay is an independent risk factor for death [21], so CVD patients are especially sensitive to the complications of pneumonia.

Three-quarters of the pneumonia cases were pneumococcal pneumonia, which was in line with earlier data [22]. For CAP, diagnosis 481 is the most sensitive, regardless of the diagnostic position [23]. When analysing the rates by sex, higher rates were observed in men, in agreement with the literature that uses the same data source [24], and are probably related to aspects of lifestyle, since CVD is associated with unhealthy lifestyle habits such as smoking, which is more common in men in Spain [25]. In addition, the second-leading cause of death in men is CVD, and the mortality rate from pneumonia is higher in men [25].

When our population was stratified by age, age was a risk factor for hospitalization and in-hospital death. This is consistent with the report that the highest percentage of deaths from pneumonia and influenza occur in people older than 75 years in Spain [25].

Between 2007 and 2010, there was an increase in HR, probably related to the fact that these were the years under study in which Spain had the greatest economic inequality in the population related to the economic crisis [26]. Specifically, in 2009, peak hospitalization occurred at all ages, a fact that may also be related to pandemic influenza A (H1N1) [27]. This increase in HR by year has also been seen in other studies of CAP in patients hospitalized in Spain [28]. There was also a decrease in 2006, probably related to the fact that this year was the hottest in the period according to the annual climatological summary of the State Meteorological Agency (Spanish Government) [29]. Finally, there was a hint of an increase in 2015 that must be given considerable attention, which makes it clear that CAP with comorbid CVD is not a thing of the past and that we should continue monitoring and studying it to protect our vulnerable population in the final stages of life.

The risk of death during hospitalization was significantly increased for men, which was consistent with the increased rate of lethality in men in our study for every age group; increased with re-admission, related to hospital stay, specifically in people older than 60 years with CAP, reducing the quality of life [30] and therefore raising the probability of being re-admitted in worse baseline condition; and finally, increased with previous admission within this period of 19 years, which can probably be explained by the improvement of health systems in our environment during this period.

This study has some limitations derived from the use of the MBDS. Reliability depends on the quality of the discharge report and clinical history, as well as on the variables of the coding process. To mitigate this variability, quality controls have been performed to evaluate the MBDS validity, and the coding process has improved since 2001. A strength is that the MBDS allowed us to carry out a complete follow-up over time for the entire population of Spain and has been used by several authors [16, 28, 31]. Specific data about bacteriological confirmation are not available within the CMBD, but a previous study assessing the accuracy of ICD-9-CM for pneumococcal pneumonia has shown high sensitivity and specificity for code 481 [23]. The use of ICD9 codes for the diagnosis of CVD and pneumonia from administrative databases could also be a limitation regarding sensitivity and specificity. However, in general, discharge diagnosis codes have proven to have a high positive predictive value (PPV) for identifying hospitalizations for common, serious infections among middle-aged and older adults [32], particularly in a setting such as Spain, where the reimbursement of physicians is not linked to the disease being treated and its severity.

The epidemiology and the rates reflected in this study of patients hospitalized with CAP and comorbid CVD reflect the need to develop and evaluate preventive measures such as vaccination for CAP in risk groups along with other vaccines, such as that against influenza, to achieve an effect in patients who are most affected by CAP, older patients, and those with CVD.

In the immunization schedule for individuals of all ages, the Spanish Ministry of Health recommends vaccination against pneumococcal infection in older people, preferably maintaining the strategy agreed upon by the Interterritorial Council of the National Health System since 2004 consisting of systematic vaccination from 65 years of age with VNP23 and not recommending periodic booster doses except in certain risk situations. The same document also refers to the VNC13 conjugate vaccine and indicates it for risk groups in the adult population and acknowledges that some autonomous regions have other alternative strategies for use of the 13v conjugate vaccine. In this sense, Madrid in 2016, and La Rioja, Asturias, Castilla y León, Galicia and Andalusia decided to apply more extensive indications of this vaccine both according to age and/or the presence of associated chronic diseases, maintaining the sequential pattern of the 13v conjugate vaccine followed by the 23v polysaccharide vaccine for risk groups [33–41].

Our results support the recommendation of experts in that an important potential to reduce CAP hospitalizations in patients with a concomitant CVD diagnosis still exists.

## Conclusions

The rates of hospitalization, mortality, and lethality increased significantly with age during the 19 years of this survey. Men had higher hospitalization and mortality rates. In-hospital death in patients with CAP and CVD was correlated with male sex, older age, and re-admission.

Community-acquired pneumonia with comorbid cardiovascular disease continues to be a major cause of hospitalization in Spain, especially in the elderly population, making it necessary to develop more preventive strategies for this group of patients.

## Data Availability

Most of the data generated or analysed during this study are included in this published article [and its additional file], although restrictions apply to the availability of some data, which were used under license for the current study, and so are not publicly available.

## Abbreviations

CAP: Community-acquired pneumonia
CVD: Cardiovascular disease
HR: Hospitalization rate
MR: Mortality rate
LR: Lethality rate
